# Temporal Dynamics of Rare and Abundant Soil Bacterial Taxa from Different Fertilization Regimes Under Various Environmental Disturbances

**DOI:** 10.1128/msystems.00559-22

**Published:** 2022-09-19

**Authors:** Yifei Sun, Xuhui Deng, Chengyuan Tao, Hongjun Liu, Zongzhuan Shen, Yaxuan Liu, Rong Li, Qirong Shen

**Affiliations:** a Jiangsu Provincial Key Lab of Solid Organic Waste Utilization, Jiangsu Collaborative Innovation Center of Solid Organic Wastes, Educational Ministry Engineering Center of Resource-saving Fertilizers, Nanjing Agricultural University, Nanjing, Jiangsu, People’s Republic of China; b Laboratory of Bio-interactions and Crop Health, Nanjing Agricultural University, Nanjing, Jiangsu, People’s Republic of China; Oak Ridge National Laboratory

**Keywords:** climate change, organic fertilization, environmental disturbance, microbial rare and abundant taxa, ecological stability

## Abstract

Global climate change has emerged as a critical environmental problem. Different types of climate extremes drive soil microbial communities to alternative states, leading to a series of consequences for soil microbial ecosystems and related functions. An effective method is urgently needed for buffering microbial communities to tackle environmental disturbances. Here, we conducted a series of mesocosm experiments in which the organic (NOF) and chemical fertilizer (NCF) long-term-amended soil microbiotas were subjected to environmental disturbances that included drought, flooding, freeze-thaw cycles, and heat. We subsequently tracked the temporal dynamics of rare and abundant bacterial taxa in NOF and NCF treatment soils to assess the efficiencies of organic amendments in recovery of soil microbiome. Our results revealed that freeze-thaw cycles and drought treatments showed weaker effects on bacterial communities than flooding and heat. The turnover between rare and abundant taxa occurred in postdisturbance succession of flooding and heat treatments, indicating that new equilibria were tightly related to the rare taxa in both NCF and NOF treatment soils. The Bayesian fits of modeling for the microbiome recovery process revealed that the stability of abundant taxa in NOF was higher than that in NCF soil. In particular, the NOF treatment soil reduced the divergence from the initial bacterial community after weak perturbations occurred. Together, we demonstrated that long-term organic input is an effective strategy to enhance the thresholds for transition to alternative states via enhancing the stability of abundant bacterial species. These findings provide a basis for the sustainable development of agricultural ecosystems in response to changing climates.

**IMPORTANCE** Different climate extremes are expected to be a major threat to crop production, and the soil microbiome has been known to play a crucial role in agricultural ecosystems. In recent years, we have known that organic amendments are an effective method for optimizing the composition and functioning of the soil microbial community and maintaining the health of the soil ecosystem. However, the effects of organic fertilization on buffering bacterial communities against environmental disturbances and the underlying mechanisms are still unclear. We conducted a series of mesocosm experiments and showed that organic fertilizers had additional capacities in promoting the soil microbiome to withstand climate change effects. Our study provides both mechanistic insights as well as a direct guide for the sustainable development of agricultural ecosystems in response to climate change.

## INTRODUCTION

Over the past century, climate change, such as drought, increased precipitation and flooding, permafrost thaw, and increased heatwave, has occurred all over the world and is becoming increasingly erratic and extreme (1). The negative effects of climate extremes on terrestrial ecosystem functions are predicted to increase (2, 3), leading to substantial changes in the cycling of carbon, nitrogen, phosphorus, and other nutrients in agricultural soil (4). These changes are associated with a marked loss of biodiversity (5), including soil microbial diversity (6). Thus, there is an increasing interest in enhancing agricultural sustainability and mitigating the effects of climate change on soil microbial communities (7), but doing so requires a better understanding of how climate change affects soil microbial ecology. Climate change influences microbial growth by altering the soil abiotic environment, including the soil moisture, temperature, and fluctuating redox, inducing unknown consequences on the stability and resilience of the soil microbiome (8, 9). Thus, soil ecosystems are sensitive to different types of climate extremes. However, most recently, researchers measured the response of microbial communities to only one disturbance, representing an oversimplification in depicting the ecological stability of communities (1). An experiment established with multifactorial climate changes can extensively identify the legacy effects of various environmental disturbances on the temporal succession of microbiota.

Under these extreme environmental condition changes, most soil microorganisms have evolved special strategies (10, 11). The strategies by which soil microorganisms respond to environmental disturbances usually depend on their genetic and physiological states (12). Thus, distinct taxon- and community-specific differences are crucial to influencing the stability and resilience of the microbiome. Traditional studies have mainly focused on the abundant members of microbial communities due to their contributions to broad functions, such as respiration and biomass (13, 14). However, recent studies have emphasized the important role of rare taxa against climate changes because of their high proportion of microbial diversity and functional redundancy (15, 16). Rare microbes may not only represent the hidden backbone of microbial communities to maintain ecosystem functions (17) but also act as a part of the microbial “seed bank” to bloom and become dominant if they adapt to certain conditions in the postdisturbance period (18). Therefore, giving more attention to both rare and abundant taxa is essential for predicting and elucidating ecosystem stability under environmental disturbances.

Increasing awareness of the impacts of climate change results in an emerging urgency to mitigate the negative consequences. Currently, direct manipulation of soil microbial communities mainly occurs through changes in land management practices (1). But the overuse of chemical fertilizers has led to negative impacts on the environment (19) and has caused biodiversity loss (20). Soil organic amendments are an effective fertilization regime that can create production and maintain ecosystem health (21). Organic matter can reportedly be used to evaluate enzyme activities and functional gene abundances related to carbon degradation under drought compared with traditional chemical fertilization (22). In our previous study, we also revealed that organic input could enhance the resistance and resilience of soil microbial communities under drought stress (23). However, the upper limit of bacterial community stability, which is enhanced by the application of organic fertilizer, is still unclear, and the mechanism of specific taxa regulating soil ecosystem stability in the organic input microbiome is still unknown.

To evaluate the effect of long-term organic inputs on the enhancement of thresholds for transition to alternative states under environmental disturbances, we applied drought (DR), flooding (FL), freeze-thaw cycles (FR), heat (HE), and ambient (AMB) treatments in soil with microbiotas from organic (NOF) versus chemical (NCF) fertilization fields. Then, we tracked the temporal dynamics of rare and abundant bacterial taxa before the start of the disturbance (initial) and at 0, 2, 40, and 170 days during the recovery period (R0, R2, R40, and R170, respectively). A quantitative model was further used to clarify the mechanism by which abundant and rare taxa affect the microbiome recovery process. We hypothesized that (i) a turnover in abundance between abundant and rare bacterial taxa may occur after disturbances, indicating the alternative states of the microbial community; and (ii) the stable bacterial community induced by organic fertilization could enhance the thresholds for transition to alternative states.

## RESULTS

### Successions of abundant and rare taxa after disturbances.

For the comparative study of abundant and rare taxa, always abundant taxa (AAT) and conditionally abundant taxa (CAT) were collectively referred to as abundant taxa, always rare taxa (ART) and conditionally rare taxa (CRT) were collectively referred to as rare taxa, and the other moderate taxa (MT) were referred to as common taxa (Table 1). Only a small fraction of the total operational taxonomic unit (OTU) number was classified as abundant (19 in NCF and 18 in NOF) but accounted for 28.45% and 32.75%, respectively, of the relative abundance of the soil bacterial communities. OTUs that were classified as rare species accounted for a large proportion of taxa (9,705 in NCF and 8,794 in NOF) and accounted for 23.58% and 20.33% of the total abundance, respectively. Common taxa contributed the most to the total abundance (nearly 50%), higher than the other two taxa.

**TABLE 1:** Detailed description of abundant, common, and rare OTUs in the initial samples

Category	NCF		NOF	
	No. (%) of OTUs	Relative abundance	No. (%) of OTUs	Relative abundance
Abundant taxa^*a*^	19 (0.18)	28.45	18 (0.19)	32.75
Common taxa^*b*^	698 (6.70)	47.97	667 (7.04)	46.92
Rare taxa^*c*^	9,705 (93.12)	23.58	8,794 (92.77)	20.33

a Abundant taxa include always abundant taxa (AAT, defined as OTUs with a relative abundance ≥1% in all initial samples) and conditionally abundant taxa (CAT, defined as OTUs with a relative abundance ≥1% in some initial samples but never <0.01% in any initial samples).

b Common taxa include moderate taxa (MT, defined as OTUs with a relative abundance between 0.01% and 1% in all initial samples).

c Rare taxa include always rare taxa (ART, defined as OTUs with a relative abundance <0.01% in all initial samples) and conditionally rare taxa (CRT, defined as OTUs with a relative abundance <0.01% in some initial samples but never ≥1% in any initial sample).

**FIG 1:**
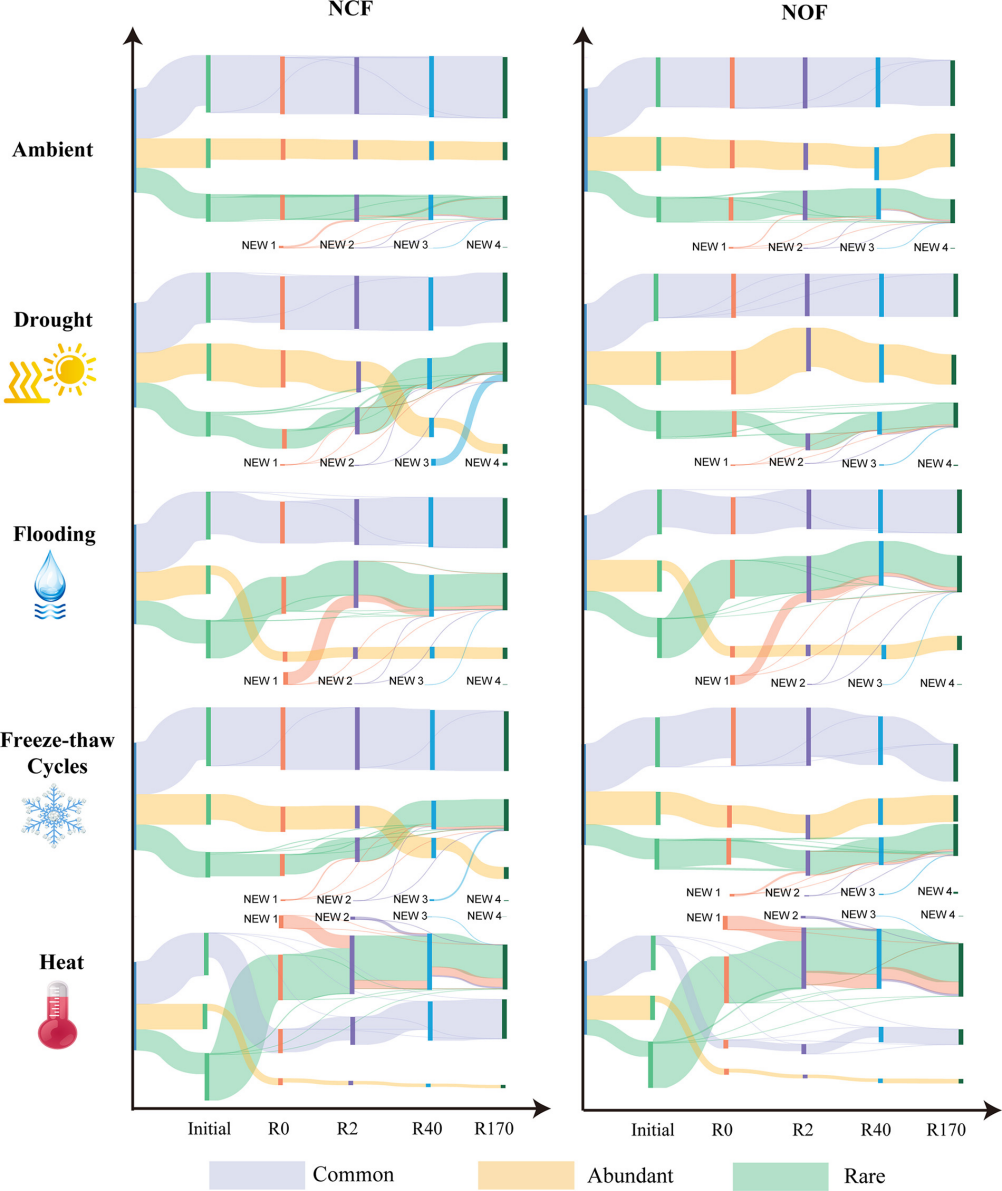
Variations in the relative abundances of abundant, common, and rare taxa tracked using Sankey plots after the initial communities experienced different disturbance treatments. The width of the stripe indicates the proportion of relative abundance, and each taxon has a distinct color. NCF, tested soil was collected from chemical fertilizer input soil; NOF, tested soil was collected from organic fertilizer input soil.

The relative abundances of abundant, common, and rare taxa within different communities revealed distinct dynamics (Fig. 1). The ratio of abundances of common, abundant, and rare taxa remained stable in the no-disturbance control (ambient). A significant influence of the relative abundances of rare and abundant taxa was observed in the FL and HE treatments, in which both the NCF and NOF communities were strongly altered. The proportion of new OTUs that appeared in FL and HE occupied approximate relative abundances of 10% and 20%, respectively. In the DR and FR treatments, the relative abundance of abundant taxa gradually decreased, while that of rare taxa increased only in the NCF treatment soil. The relative abundance of common taxa was steady in most situations but was only altered by HE treatment.

### Variations in alpha and beta diversity compared with the initial community.

The log response ratios (LRRs) of the Shannon index between the initial and other samples following the experimental design were calculated to show the diversity variations (see Fig. S1 in the supplemental material). In the DR, FR, and HE treatments, the LRRs of rare taxa were always higher than those of abundant taxa. However, the values of rare taxa were significantly lower than those of abundant taxa in the FL treatment (*t* test, *P < *0.05). In metrics of fertilization regimes, the LRRs of common and rare taxa in the DR treatment, common taxa in the FL treatment, and abundant and common taxa in the HE treatment in NOF were significantly higher than those in NCF (*t* test, *P < *0.05).

10.1128/msystems.00559-22.1 FIG S1 Pairwise log response ratios (LRRs) of the Shannon index of abundant, common, and rare taxa between initial samples and other samples following the experimental design. DR, drought treatment; FL, flooding treatment; FR, freeze-thaw cycle treatment; HE, heat treatment; NCF, tested soil was collected from chemical fertilizer input soil; NOF, tested soil was collected from organic fertilizer input soil. *, *P* < 0.05; **, *P* < 0.01; ***, *P* < 0.001, based on *t* test comparison between two communities, respectively. Download FIG S1, TIF file, 1.3 MB. Copyright © 2022 Sun et al. 2022 Sun et al. https://creativecommons.org/licenses/by/4.0/ This content is distributed under the terms of the Creative Commons Attribution 4.0 International license.

**FIG 2:**
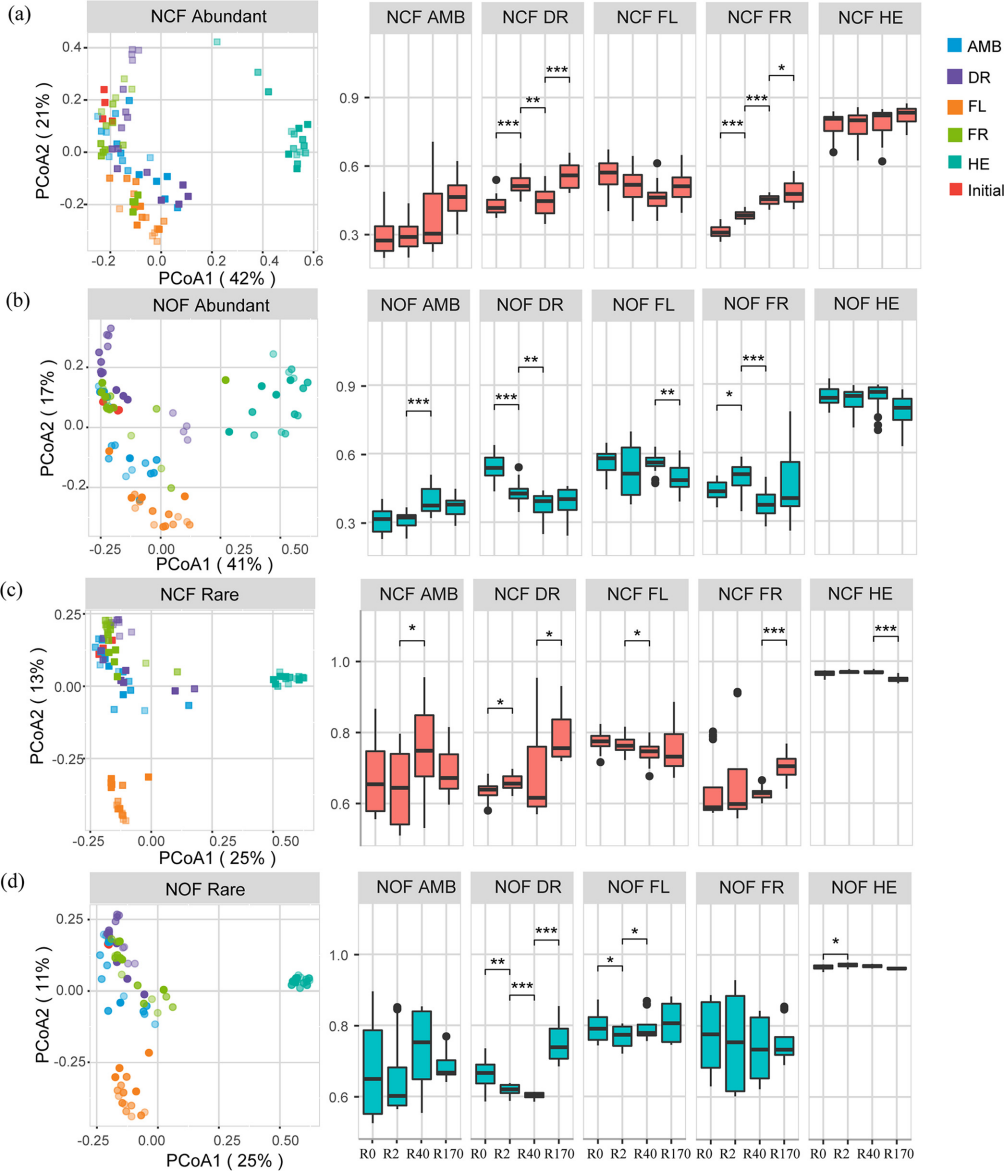
Variations in microbial taxonomic structure and Bray-Curtis dissimilarity with initial samples under different disturbances in the community of NCF abundant taxa (a), NOF abundant taxa (b), NCF rare taxa (c), and NOF rare taxa (d). Colors indicate different disturbance treatments. Squares and circles represent the NCF and NOF communities, respectively. The pairwise dissimilarity between communities at neighboring time points was calculated based on a *t* test comparison; *, *P* < 0.05; **, *P* < 0.01; ***, *P* < 0.001, respectively. AMB, ambient; DR, drought treatment; FL, flooding treatment; FR, freeze-thaw cycle treatment; HE, heat treatment; NCF, tested soil was collected from chemical fertilizer input soil; NOF, tested soil was collected from organic fertilizer input soil.

A significant influence of disturbances on both abundant, common, and rare community structures was observed in the two soil systems based on the principal-coordinate analysis (PCoA) (Fig. 2 and Fig. S2). Both disturbance type and time significantly altered all community structures (permutational multivariate analysis of variance [PERMANOVA], *P < *0.01) (Table S1). For abundant taxa, the community Bray-Curtis dissimilarity of the DR and FR treatments in NCF increased over time, while that in NOF had a continuous decrease from R0 to R170, as indicated in Fig. 2a and b. Based on the similarity percentage (SIMPER) analysis, rare taxa revealed a greater contribution to the overall dissimilarity of bacterial communities induced by disturbances than abundant taxa (Fig. S3). Rare taxa accounted for 22.6% to 55.7% of the variations in the overall microbial communities, while abundant taxa accounted for approximately 14.0% to 33.9%. The contributions of abundant taxa in the NOF treatment soil were significantly higher than those in the NCF treatment soil (*t* test, *P < *0.05) (Fig. S3e).

10.1128/msystems.00559-22.2 FIG S2 Variations in microbial taxonomic structure and Bray-Curtis dissimilarity with initial samples under different disturbances in the community of NCF common taxa (a) and NOF common taxa (b). Colors indicate different disturbance treatments. Squares represent the communities in NCF, and circles represent the communities in NOF. The pairwise dissimilarity between communities at neighboring time points was calculated based on *t* test comparison; *, *P* < 0.05; **, *P* < 0.01; ***, *P* < 0.001. AMB, ambient; DR, drought treatment; FL, flooding treatment; FR, freeze-thaw cycle treatment; HE, heat treatment; NCF, tested soil was collected from chemical fertilizer input soil; NOF, tested soil was collected from organic fertilizer input soil. Download FIG S2, TIF file, 1.6 MB. Copyright © 2022 Sun et al. 2022 Sun et al. https://creativecommons.org/licenses/by/4.0/ This content is distributed under the terms of the Creative Commons Attribution 4.0 International license.

10.1128/msystems.00559-22.3 FIG S3 Contributions (%) of NCF abundant taxa (a), NCF rare taxa (b), NOF abundant taxa (c), and NOF rare taxa (d) to the overall dissimilarity of microbial communities based on similarity percentage (SIMPER) analysis. Colors indicate different disturbance treatments. Alpha from light to dark indicates time dynamics from R0 to R170. (e) Boxplot represents the comparison of contributions in NCF with NOF soil system. AMB, ambient; DR, drought treatment; FL, flooding treatment; FR, freeze-thaw cycle treatment; HE, heat treatment; NCF, tested soil was collected from chemical fertilizer input soil; NOF, tested soil was collected from organic fertilizer input soil. Download FIG S3, TIF file, 1.9 MB. Copyright © 2022 Sun et al. 2022 Sun et al. https://creativecommons.org/licenses/by/4.0/ This content is distributed under the terms of the Creative Commons Attribution 4.0 International license.

10.1128/msystems.00559-22.7 TABLE S1 Permutation multivariate analysis of variance (PERMANOVA) results for the bacterial community structure of different taxa. Values in the table represent the estimation of the variance component (*R*^2^) and the level of significance for PERMANOVA. *P* values were calculated based on 999 permutations. NCF, tested soil was collected from chemical fertilizer input soil; NOF, tested soil was collected from organic fertilizer input soil. Download Table S1, DOCX file, 0.02 MB. Copyright © 2022 Sun et al. 2022 Sun et al. https://creativecommons.org/licenses/by/4.0/ This content is distributed under the terms of the Creative Commons Attribution 4.0 International license.

### Disturbance-responsive individuals within and between compartments.

**FIG 3:**
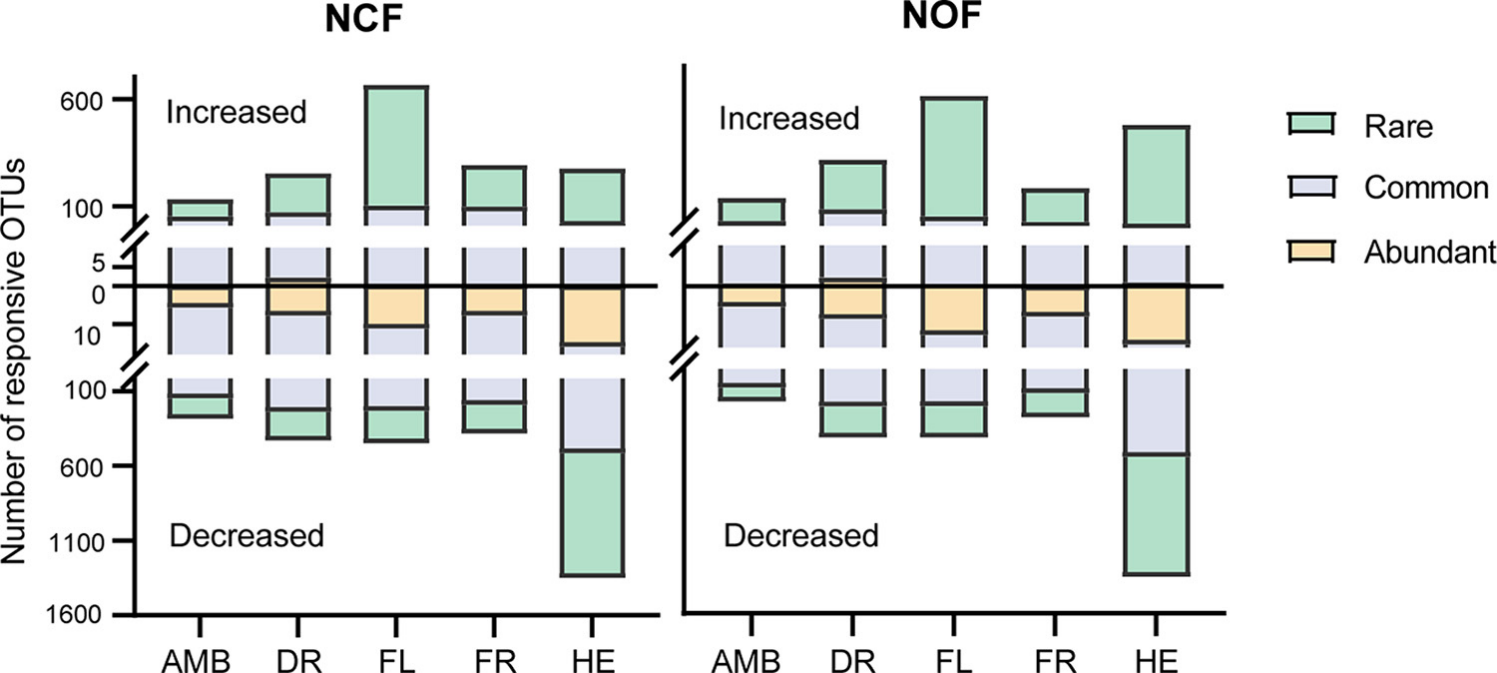
Numbers of disturbance-responsive OTUs detected through negative binomial models in abundant, common, and rare taxa. Only individual OTUs with *P*_FDR_ values of <0.05 and log_2_ fold change of >1 were counted. The top half of the bar plots showed upregulated OTUs, and the bottom half showed downregulated OTUs. AMB, ambient; DR, drought treatment; FL, flooding treatment; FR, freeze-thaw cycle treatment; HE, heat treatment; NCF, tested soil collected from chemical fertilizer input soil; NOF, tested soil collected from organic fertilizer input soil.

The number of responsive OTUs (false-discovery rate–adjusted *P* value [*P*_FDR_] < 0.05, log_2_ fold change > 1) is shown in Fig. 3. AMB treatment had the smallest number of responsive OTUs, while the greatest number of responsive OTUs was observed in the HE treatment. In general, the rank of perturbation strength of disturbance was HE > FL > DR > FR > AMB. The number of disturbance-responsive OTUs belonging to rare taxa was highest, and the average fold change of rare taxa was higher than that of abundant taxa (Fig. S4). The OTUs exhibiting enrichment belonged mainly to the phyla Actinomyces, Acidobacterium, Firmicutes, and Proteobacteria (Fig. S5).

10.1128/msystems.00559-22.4 FIG S4 Disturbance-responsive OTUs detected through negative binomial models in abundant, common, and rare taxa. Only individual OTUs with *P*_FDR_ values of <0.05 and log_2_ fold change >1 were colored in the volcano plot. The top half of the volcano plots showed upregulated OTUs, and the bottom half showed downregulated OTUs. The inset doughnut plots display the taxa composition of overall upregulated or downregulated OTUs. The bar plots on the right side present the average fold change of responsive OTUs belonging to abundant, common, and rare taxa, separately. The color represents the different taxa; light gray indicates OTUs that are not significantly different or have a fold change <2. AMB, ambient; DR, drought treatment; FL, flooding treatment; FR, freeze-thaw cycle treatment; HE, heat treatment; NCF, tested soil collected from chemical fertilizer input soil; NOF, tested soil collected from organic fertilizer input soil. Download FIG S4, TIF file, 1.5 MB. Copyright © 2022 Sun et al. 2022 Sun et al. https://creativecommons.org/licenses/by/4.0/ This content is distributed under the terms of the Creative Commons Attribution 4.0 International license.

10.1128/msystems.00559-22.5 FIG S5 Classification of the significantly enriched OTUs detected through negative binomial models in R0 and R170 timepoint samples. The size of the tiles indicates the number of OTUs in particular taxa, while the color represents the different taxa. A, abundant taxa; C, common taxa; R, rare taxa; AMB, ambient; DR, drought treatment; FL, flooding treatment; FR, freeze-thaw cycle treatment; HE, heat treatment; NCF, tested soil was collected from chemical fertilizer input soil; NOF, tested soil was collected from organic fertilizer input soil. Download FIG S5, TIF file, 1.6 MB. Copyright © 2022 Sun et al. 2022 Sun et al. https://creativecommons.org/licenses/by/4.0/ This content is distributed under the terms of the Creative Commons Attribution 4.0 International license.

### Modeling microbiome recovery using a stability landscape framework.

**FIG 4:**
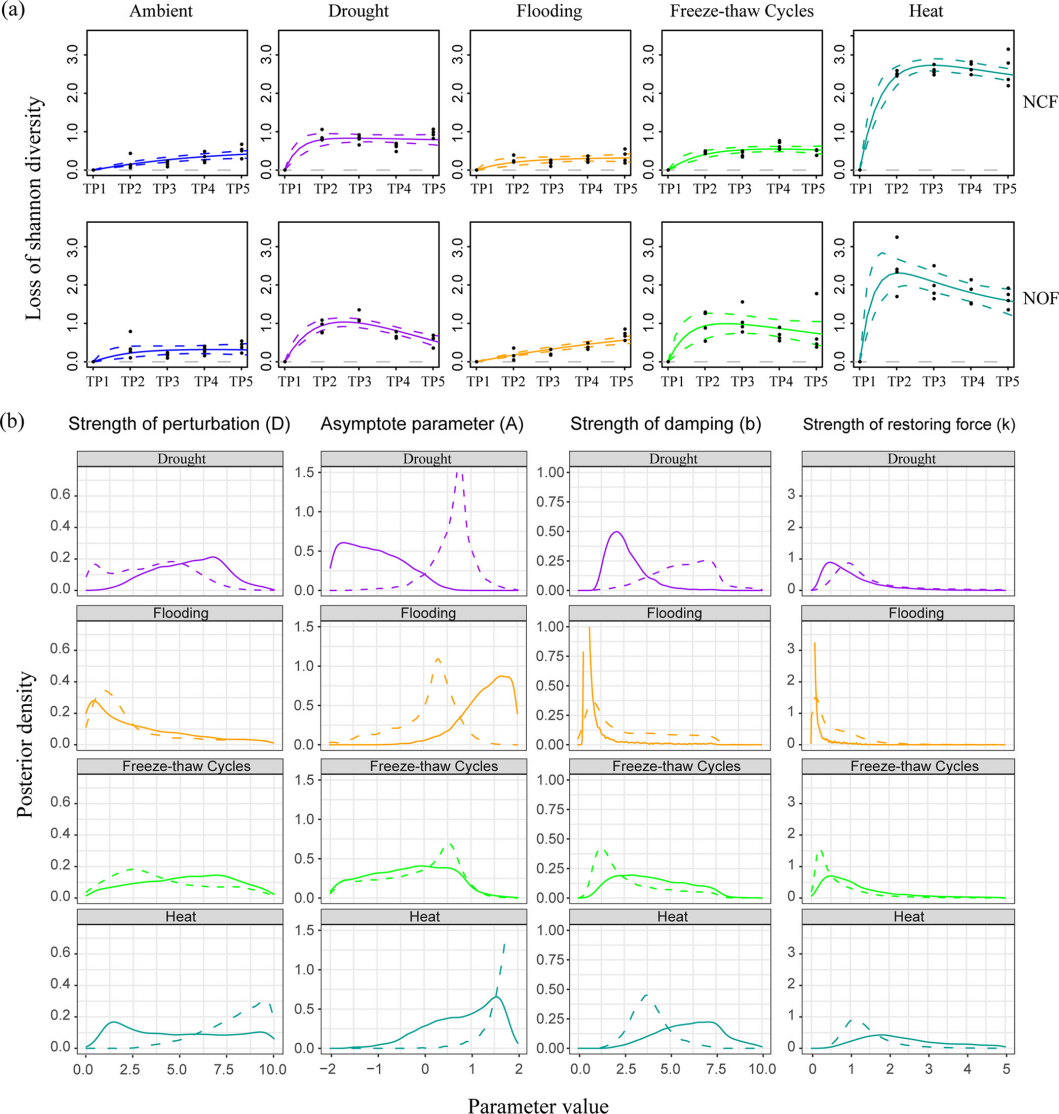
Model of the stability landscape for the abundant taxa in the NCF and NOF microbiomes after disturbances. (a) Temporal dynamics of diversity recovery. Bayesian fits for initial samples that experienced no stress, drought, flooding, freeze-thaw cycles, and heat (*n* = 4). The mean Shannon diversity for the abundant taxa in each sample (black point) and median and 95% credible interval from the posterior distribution (bold and dashed colored lines, respectively) are shown. (b) Posterior parameter estimates for the model. The posterior distributions from Bayesian fits of the model from NCF (dashed) and NOF (solid) microbiomes of treatments that experienced drought (purple), flooding (orange), freeze-thaw cycles (green), and heat (cyan). TP1, -2, -3, -4, and-5 correspond to time point initial, R0, R2, R40, and R170, respectively. NCF, tested soil was collected from chemical fertilizer input soil; NOF, tested soil was collected from organic fertilizer input soil.

We captured the same variation pattern in different disturbance treatments, with diversity decreasing before the end of stress and then recovering gradually (Fig. 4a), but the recovery rate and range of variation in diversity for abundant taxa in NCF and NOF were different. The Bayesian fit line in NOF was more curing than that in NCF, while this phenomenon in the model of rare taxa was not observed (Fig. S6a).

10.1128/msystems.00559-22.6 FIG S6 Model of the stability landscape for the rare taxa in the NCF and NOF microbiomes after disturbances. (a) Temporal dynamics of diversity recovery. Bayesian fits for initial samples that experienced no stress, drought, flooding, freeze-thaw, cycles and heat (*n* = 4). The mean Shannon diversity for rare taxa in each sample (black point) and median and 95% credible interval from the posterior distribution (bold and dashed colored lines, respectively) are shown. (b) Posterior parameter estimates for the model. The posterior distributions from Bayesian fits of the model to empirical data from NCF (dashed) and NOF (solid) microbiomes of treatments that experienced drought (purple), flooding (orange), freeze-thaw cycles (green), and heat (cyan). TP means the timepoint, and TP1, -2, -3, -4, and -5 correspond to the initial, R0, R2, R40, and R170, respectively. NCF, tested soil was collected from chemical fertilizer input soil; NOF, tested soil was collected from organic fertilizer input soil. Download FIG S6, TIF file, 2.8 MB. Copyright © 2022 Sun et al. 2022 Sun et al. https://creativecommons.org/licenses/by/4.0/ This content is distributed under the terms of the Creative Commons Attribution 4.0 International license.

The posterior distributions allowed us to compare the values of various parameters from Bayesian fits of the model based on different strengths of perturbations, taxon types, and soil microbiomes. The posterior probability distribution is a way of visualizing the uncertainty in parameter values after model fitting (a tighter peak indicates more certainty about the parameter value). Because the sum under the distribution is defined as being equal to one, the scale of the *y* axis depends on the range of the *x* axis; that is, it has no absolute meaning. The peaks of model parameters for abundant taxa in NCF and NOF did not overlap, providing evidence of different stabilities in abundant taxa (Fig. 4). For abundant taxa, the median (95% credible interval) asymptote parameters of the DR treatment were 0.70 (−0.19 to 1.23) in NCF and −1.13 (−1.91 to 0.02) in NOF, and those of the FR treatment were 0.03 (−1.70 to 0.91) in NCF and −0.28 (−1.74 to 0.94) in NOF (Table 2). The posterior estimates for the asymptotic parameter of the DR and FR treatments were positively skewed in NOF, while those in NCF were negatively skewed (Fig. 4b), suggesting completely different community transition states. In contrast, in the model parameters for rare taxa, there was no significant difference between NCF and NOF regardless of the disturbance treatments (Fig. S6b and Table S2).

**TABLE 2:** Medians and 95% credible intervals for all model parameters of abundant taxa

Abundant microbiome^*a*^	Stress	D		A		Phi1		Phi2	
		Median	95% CI	Median	95% CI	Median	95% CI	Median	95% CI
NCF	DR	3.63	0.28 to 6.65	0.70	−0.19 to 1.23	−1.46	−1.95 to 0.99	1.69	0.94 to 1.97
	FL	1.63	0.25 to 8.33	0.24	−1.25 to 0.89	−1.59	−1.96 to −0.38	0.62	−1.23 to 1.91
	FR	3.47	0.63 to 8.72	0.03	−1.70 to 0.91	−1.48	−1.95 to −0.27	0.50	−0.55 to 1.85
	HE	8.27	4.69 to 9.86	1.80	1.14 to 1.99	−1.00	−1.24 to −0.32	1.22	0.65 to 1.69
NOF	DR	5.62	2.38 to 8.31	−1.13	−1.91 to 0.02	−0.84	−1.73 to −0.01	0.61	−0.05 to 1.36
	FL	2.08	0.09 to 8.27	1.41	0.37 to 1.93	−1.77	−1.97 to −1.28	−1.18	−1.80 to 0.97
	FR	5.47	1.10 to 9.12	−0.28	−1.74 to 0.94	−1.33	−1.93 to 0.18	1.21	0.05 to 1.92
	HE	4.60	1.00 to 9.61	0.94	−0.51 to 1.76	−0.47	−1.29 to 1.22	1.65	0.92 to 1.97

a NCF, tested soil was collected from chemical fertilizer input soil; NOF, tested soil was collected from organic fertilizer input soil; AMB, ambient; DR, drought treatment; FL, flooding treatment; FR, freeze-thaw cycle treatment; HE, heat treatment; D, strength of perturbation; A, asymptote parameter; Phi1, ϕ1; Phi2, ϕ2.

### The new equilibria are tightly related to the ratio of rare and abundant taxa.

**FIG 5:**
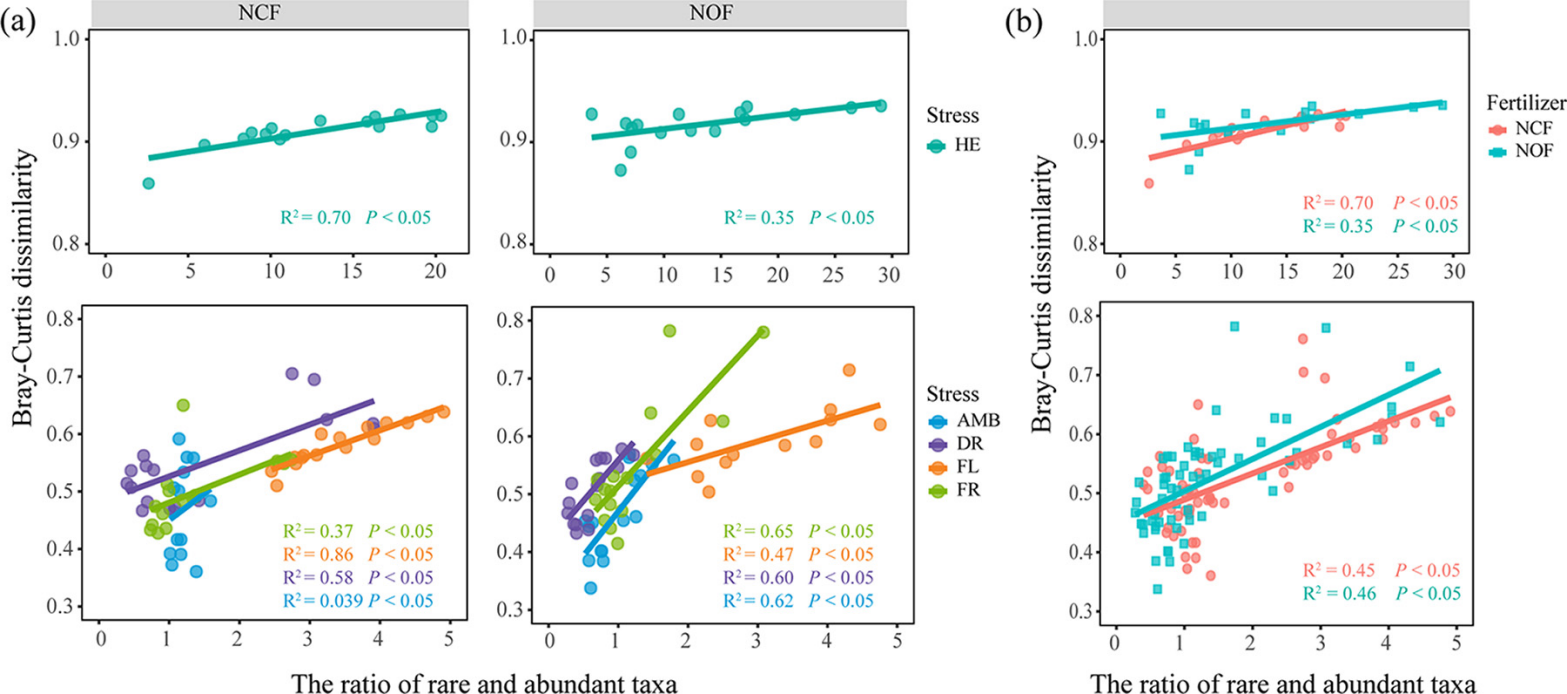
Linear regression between the ratio of rare and abundant taxa based on relative abundance and overall community Bray-Curtis dissimilarity in different disturbance treatments (a) and different fertilization regimes (b). HE treatment was plotted by itself because of the distinct values of the *y* axis and *x* axis. AMB, ambient; DR, drought treatment; FL, flooding treatment; FR, freeze-thaw cycle treatment; HE, heat treatment; NCF, tested soil collected from chemical fertilizer input soil; NOF, tested soil collected from organic fertilizer input soil.

A linear regression analysis between the ratio of the relative abundance of rare and abundant taxa and the Bray-Curtis dissimilarity of the overall community was performed (Fig. 5). Except for the AMB in NCF, all regressions presented a significant positive relationship (*R*^2^ > 0, *P < *0.05). In the metric of four disturbances, the slope of fit lines for all kinds of stress in NCF was similar, while the fit line for DR treatment in NOF was flatter (*R*^2^ = 0.47, *P < *0.05) than the other lines. In the metric of fertilization treatments, the *R*^2^ values in NCF (*R*^2^ = 0.45, *P < *0.05) and NOF (*R*^2^ = 0.46, *P < *0.05) were almost the same.

## DISCUSSION

The responses of rare and abundant bacterial communities were distinct under various disturbances. We revealed that the log response ratios of alpha diversity in rare taxa declined less than those in abundant taxa in the drought, freeze-thaw cycles, and heat treatments, indicating less microbial taxonomic diversity loss (24). A possible explanation for this is that the initial rarity of rare taxa is the result of fitness trade-offs, for instance, when stress resistance comes at the cost of a low growth rate (25). Slow-growing rare species might not reach a high density but may persist well to overcome stressful conditions to maintain the diversity of rare taxa. For the flooding treatment, we found an opposite trend for diversity variation in abundant and rare taxa. Flooding is a homogenous process that fills soil pores with water and removes soil oxygen (26), unlike other disturbances that create patchy resources and isolate ecological niches. Thus, the higher the diversity in rare taxa, the more it will be lost under flooding conditions. Although the alpha diversity seemed more stable in the rare community, greater community dissimilarity (beta diversity) and more contributions to the overall community dissimilarity after perturbations than that of the abundant community were observed. These results agree with the findings of other studies in which the rare bacterial community was more sensitive to environmental changes than the abundant bacterial community in the beta diversity dimension (27). The sensitivity of the rare taxa could be explained by their narrow environmental breadths to environmental changes (28). Thus, we speculate that rare taxa act as reservoirs that can rapidly respond to environmental change (18), as conditionally rare taxa are members of a seed bank that can bloom under favorable conditions (29).

A turnover of relative abundance appeared between abundant and rare taxa in our study. Although only a few rare taxa significantly increased during stress in a previous study (30), we found that rare taxa gradually became dominant in the postdisturbance succession. Organism abundance is an outcome of its balance of lifestyle and the current environmental conditions. Rare species have been hypothesized to be on the more copiotrophic side of the spectrum from oligotrophic to copiotrophic lifestyles (31), and they can exhibit fast growth under high nutrient concentrations (32). They might be rare in initial stable environments but could be selected for after disturbances. The increase in many rare taxa after environmental changes indicates that these taxa might be not only copiotrophs but also opportunistic. In addition, rare species maintaining important ecosystem functions after changes in environmental conditions are called the insurance effect (33). Increasing the abundance of rare taxa is supposed to be an important mechanism (34). Some evidence suggests that the changes in rare taxa might become permanent (35), and these variations must be considered because they could cause a positive or negative effect. Previous studies have already observed that rare members could harbor large functional potential in relation to maize yield under changing climate regimes (17), while some rare pathogens can also be strongly affected, such as the fungal soil pathogen Coccidioides immitis in California, which causes coccidioidomycosis (also known as valley fever) and benefits from an extreme shift in precipitation (36).

When the organic amended bacterial community responded to disturbances of drought and freeze-thaw cycles, a turnover of relative abundances between abundant and rare taxa did not occur, and community dissimilarity tended to decrease in the recovery stage (Fig. 1 and 2). On one hand, the difference in response to various disturbances may contribute to the different perturbation strengths. As disturbances vary tremendously with respect to their influences on the soil abiotic properties, it is difficult to generalize the same response of soil microbiomes across different environmental disturbances (36). Drought and freezing affect the state of water in soils and its bioavailability (37). Following the less-liquid water in soil pores, microorganisms may enter a dormant state until the environment is rewetted and active again (38, 39), and this process is reversible. Flooding treatment can allow soil pores to become water filled and result in a community dominated by anaerobic microorganisms (26). The direct effect of soil heating on microorganisms includes cell death due to protein denaturation and cell lysis, resulting in a permanent reduction in microbial biomass (40).

On the other hand, organic input manipulates specific taxa to enhance overall community stability against environmental disturbances. We revealed that a new alternate stable state of community was always established around the blooming of rare taxa, but the abundant taxa determined if the whole community tended to recover to the original state after the disturbance of weak strength (drought or freeze-thaw cycles). Similar to the posterior estimates for the asymptote parameter of abundant taxa in organic fertilizer treatment, the negative skew in the drought and freeze-thaw cycles treatments indicates gradual recovery to the initial state of the community. We chose the diversity value as a proxy because it is commonly used as a summary statistic in microbiome analyses, and higher diversity has previously been generally associated with soil (41) or gut microbiome health (42, 43). Moreover, variations in abundant taxa in organic fertilization treatment were important to the overall dissimilarity of microbial communities induced by environmental disturbances compared with that in chemical fertilization treatment, which highlights the organic input manipulating the abundant taxa to enhance overall community stability.

**FIG 6:**
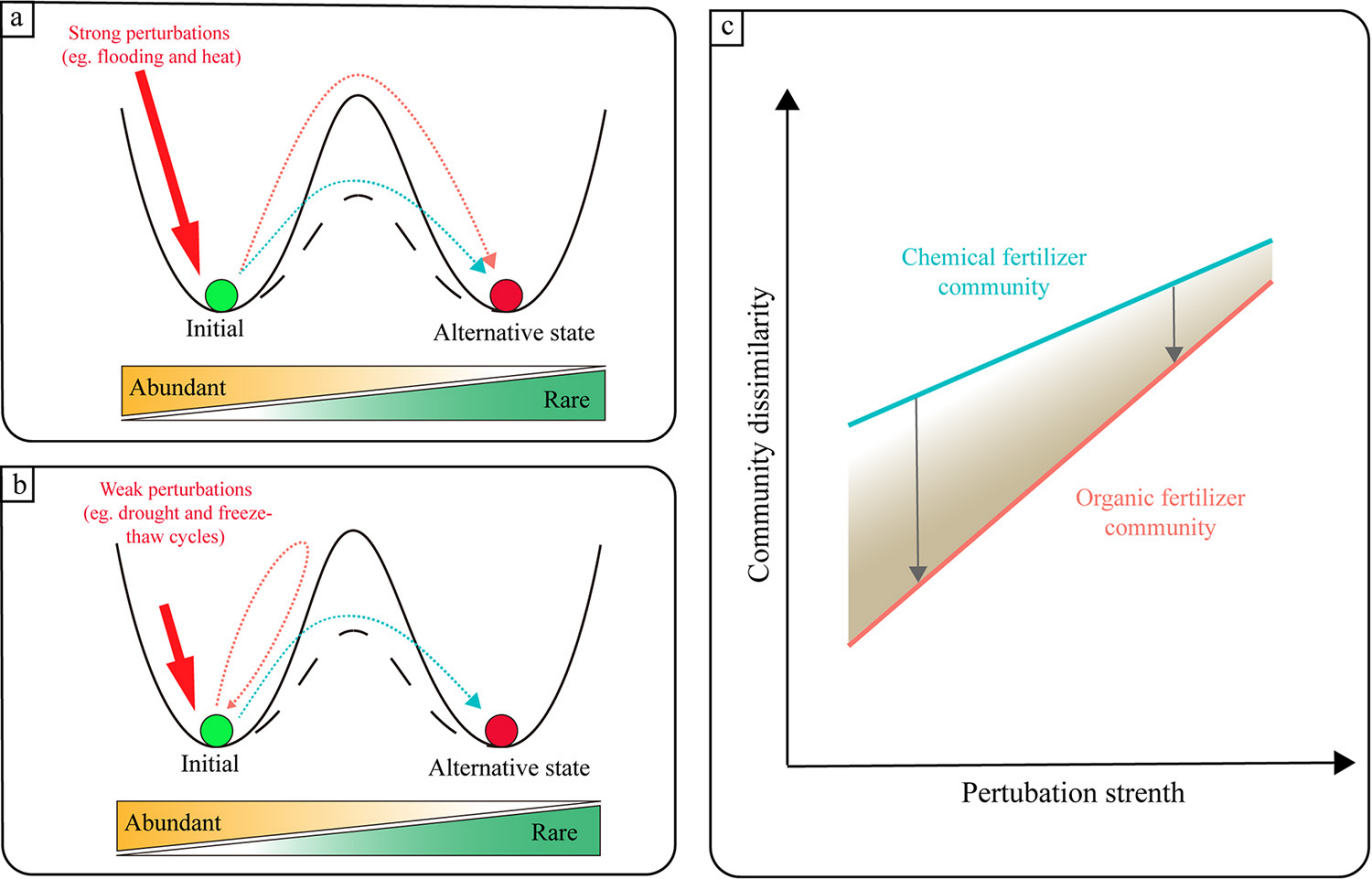
Conceptual diagrams showing our hypotheses in this study. The strong perturbations (a) and weak perturbations (b) were distinguished in the stability landscape framework for the microbiome postdisturbance succession. The ball in the basin represents the “state” of the system. The red dashed arrow represents the possible response of a system with high damping, while the blue dashed arrow represents a system with low damping. The gradient color of the bar below represents the dominant taxa in the two states. (c) Organic input can decrease the community dissimilarity with the initial community compared with chemical fertilization regimes, and this effect can be weakened by an enhanced perturbation strength.

Based on a popular schematic picture taken from classical ecology, our findings are displayed using a “ball and cup” model (44). Several studies have supported the possibility of transfer to an alternative microbiome state after a disturbance in the soil, oral, or gut microbiomes (45, 46). Our research demonstrated that the key to whether the microbial community will enter a new alternative state in the postdisturbance succession is the perturbation strength (environmental disturbance type) and the damping of a system (stability of the soil ecosystem) (Fig. 6a and b). When strong disturbances are applied, the soil ecosystems with either high or low stability will transfer into alternative states; when weak disturbances are applied, systems with low stability (such as chemical fertilization treatment) will transfer into alternative states, while systems with high stability (such as organic fertilization treatment) will return to the initial state. Different taxa components of the community are tightly related to this ecological process. Organic amendments mainly increase abundant taxa stability to decrease divergence from the initial community. However, the effects of organic input could be weakened by an enhanced perturbation strength (Fig. 6c). When the initial state of the microbiome has slipped into the alternative state, the new equilibria of the community would assemble around the activity of rare taxa.

### Conclusions.

The temporal dynamics of rare and abundant soil bacterial taxa from different fertilization regimes suffering various environmental disturbances were depicted in this study. The rare taxa had lower log response ratios of Shannon diversity but contributed a higher proportion to the overall dissimilarity of bacterial communities than the abundant taxa. The rare taxa gradually became dominant in communities, which may indicate that new equilibria are tightly related to the rare taxa. Organic amendments are an effective strategy for enhancing the stability of abundant taxa and inducing the community to recover to the initial state after weak perturbations occur. Our study has no gradients for each environmental disturbance, and it remains to be studied if these results would be consistent under broad conditions. Still, these findings provide both mechanistic insights as well as a direct guide for the sustainable development of agricultural ecosystems in response to changing climates.

## MATERIALS AND METHODS

### Experimental design.

Mesocosm experiments were constructed with the soils containing microbiota from organic (NOF) and chemical (NCF) fertilization fields, and the tested soils’ collection, inoculation, and cultivation have been described in detail in our previous study (23). Plastic tissue culture bottles (350 mL) that had a 0.22-μm filter membrane were used to prevent cross contamination with microbes while allowing gas and water vapor exchange. These soils were exposed to four disturbances (two moisture-related stresses, including drought and flooding, and two temperature-related stresses, including freeze-thaw cycles and heat) and one control treatment with four technical replicates. Details of the treatments are described in Table 3. The standard environment state was set as moisture of 17% for the soil (50% of field capacity) and 28°C temperature (simulating in growing season) for cultivation in the incubators. All disturbance treatments were linked to ecologically relevant conditions and simulated a variety of different stressors. The drought treatment was the same as our previous study (23). The flooding treatment consisted of 5 cm of water above the soil surface to make sure the conditions were anoxic or anaerobic (26). The range of minimum and maximum temperatures used in freeze-thaw cycle treatment was set according to most similar experiments (47). In heat treatment, soil was incubated at 45°C with daily adjustment of the moisture to the standard level. All disturbances were applied for 80 days in a constant manner. After 80 days, the disturbances were stopped, and all treatments started to recover for another 170 days. As we paid attention to both short-term and long-term recovery, the sampling time interval was quite large. Samples were taken before the start of the disturbance (initial time) and at 0, 2, 40, and 170 days during the recovery period (R0, R2, R40, and R170, respectively). The sampling time was more intensive during early recovery because the bacterial community tended to be stable following prolonged recovery time (46). Seventy-two samples of ambient and drought treatments came from our previous study (23) (2 fertilizer treatments × 2 climate change treatments × 4 time points × 4 replicates + 8 initial samples). Five grams of soil in each bottle of other treatments was sampled, consisting of another 96 samples (2 fertilizer treatments × 3 climate change treatments × 4 time points × 4 replicates). After sieving through a 2-mm sieve, all samples were stored at −80°C for soil DNA extraction.

**TABLE 3:** Details of different disturbance treatments

Abbreviation	Name	Stress description	Recovery description
AMB	Ambient	No addition	No addition
DR	Drought	Without lids and air-dried by an electric fan	Rewetting to 50% field capacity moisture
FL	Flooding	5 cm of deionized water above the soil surface	Discharge redundant gravitational water and decrease to 50% field capacity moisture
FR	Freeze-thaw cycles	Each cycle consisted of freezing at −20°C for 24 h, followed by thawing and incubation at 28°C for 48 h	28°C in an incubator
HE	Heat	45°C in an incubator	28°C in an incubator

### Soil DNA extraction and Illumina MiSeq sequencing.

Total soil genomic DNA was extracted from 0.25 g of soil using the PowerSoil DNA isolation kit (Mobio Laboratories, Carlsbad, CA, USA) according to the manufacturer’s instructions. The quality and quantity of DNA were determined using a NanoDrop 2000 spectrophotometer (Thermo Scientific, Waltham, MA, USA). Bacterial sequencing libraries were constructed according to previously described protocols (48, 49). Investigation of bacterial communities was based on paired-end amplicon sequencing of the 16S rRNA gene on an Illumina MiSeq PE 250 platform at Magigene Biotechnology Co., Ltd. (Guangdong, China). Amplification of the V4-V5 hypervariable regions of the 16S rRNA genes was performed using the general bacterial primers 515F (5′-GTGCCAGCMGCCGCGGTAA-3′) and 907R (5′-CCGTCAATTCMTTTRAGTTT-3′) (50).

The raw split sequences were merged using USEARCH (version 11.0) (51). After trimming the adaptors and primer sequences, quality filtering was performed using VSEARCH (version 2.15.0) (52). Then, the remaining reads were clustered into operational taxonomic units (OTUs) at a 97% similarity identity level. Finally, a representative sequence for each OTU was selected and classified using the RDP classifier against the RDP 16S rRNA database (53). Sequences were randomly subsampled to 67,899 reads per sample for 16S rRNA gene sequences. The relative abundance of a given taxonomic group per sample was calculated as the number of sequences affiliated with that group divided by the total number of sequences.

### Definition of abundant and rare taxa.

Following recent studies (17, 54), the definition of abundant and rare taxa in the initial samples depended on the cutoff level of relative abundance, and 0.01% and 1% were set as the thresholds for rare and abundant OTUs, respectively. The new OTUs that appeared in later samples were identified as rare taxa in the initial samples. All OTUs were classified into six categories, always abundant taxa (AAT), with a relative abundance ≥1% in all initial samples; conditionally abundant taxa (CAT), with a relative abundance ≥0.01% in all initial samples and ≥1% in some initial samples; always rare taxa (ART), with a relative abundance of <0.01% in all initial samples; conditionally rare taxa (CRT), with a relative abundance of <0.01% in some initial samples but never ≥1% in any initial sample; moderate taxa (MT), with a relative abundance between 0.01% and 1% in all initial samples; and conditionally rare and abundant taxa (CRAT), with a relative abundance ranging from rare (<0.01%) to abundant (≥1%). Then, for the comparative study of abundant and rare taxa, AAT and CAT collectively referred to abundant taxa, and the rare taxa consisted of ART and CRT. No OTUs were classified as CRAT in our study, so the common taxa only included MT.

### Statistical analysis.

All statistical analyses were performed using the R software program (version 3.6.0). The alpha diversity variations of abundant, common, and rare bacterial communities were shown by the LRRs of the Shannon index between initial samples and other samples following the experimental design (55). Unpaired *t* tests and one-way analysis of variance (ANOVA) were performed to determine significant differences between the LRRs of alpha diversity. All statistical tests performed in this study were considered significant at *P* values of <0.05. A principal-coordinate analysis (PCoA) based on a Bray-Curtis dissimilarity matrix was also performed and plotted to explore the differences in bacterial community structures across all soil samples. A permutational multivariate analysis of variance (PERMANOVA) was conducted to evaluate the effects of fertilization regimes and disturbances on the soil bacterial community. SIMPER was used to assess which subcommunity was primarily responsible for an observed difference between groups of samples (56). All of the above analyses were performed using the vegan package (57).

The DESeq2 package (58) was used to examine the effects of experimental factors on the abundance of individual OTUs. Within each disturbance (AMB, DR, FL, FR, and HE) and each fertilization regime (NCF and NOF), pairwise Wald tests were performed to compare individual time point samples (R0, R2, R40, and R170) against initial samples. For each contrast, effect size shrinkage was performed using the lfcShrink() function. After using the false-discovery rate (Benjamini-Hochberg procedure) to adjust *P* values, only individual OTUs with *P* values of <0.05 and log_2_ fold change >1 were examined in this study.

We fit our data into a quantitative model based on the stability landscape concept (46). Briefly, this analytical impulse-response model has assumptions with four parameters, *b* (the damping of the system), *k* (the strength of the restoring force), *D* (how strong the perturbation is), and *A* (new value of equilibrium diversity). This model was fitted in a Bayesian framework of the effects of four disturbances on the abundant and rare bacterial communities in the NCF and NOF microbiomes. We chose Shannon diversity metrics as a proxy for the summary statistic in the microbiome analyses. After modeling the dynamics of recovery for the abundant and rare communities after disturbances in NCF and NOF, the posterior distribution of parameters for the models was also evaluated. Finally, a linear regression analysis relating the ratio of rare and abundant taxa based on relative abundance to overall community Bray-Curtis dissimilarity was fitted using geom_smooth with the lm function in ggplot2.

### Data availability.

The raw sequence data for the 16S rRNA gene V4-V5 region of all samples were submitted to the NCBI Sequence Read Archive (SRA) database (http://www.ncbi.nlm.nih.gov/) under BioProject accession number PRJNA817056. The R code supporting the findings presented here is available from GitHub (https://github.com/dxh7844/R_analysis_code).

10.1128/msystems.00559-22.8 TABLE S2 Medians and 95% credible intervals for all model parameters of rare taxa. AMB, ambient; DR, drought treatment; FL, flooding treatment; FR, freeze-thaw cycle treatment; HE, heat treatment; NCF, tested soil was collected from chemical fertilizer input soil; NOF, tested soil was collected from organic fertilizer input soil; D, strength of perturbation; A, asymptote parameter; Phi1, ϕ1; Phi2, ϕ2. Download Table S2, DOCX file, 0.02 MB. Copyright © 2022 Sun et al. 2022 Sun et al. https://creativecommons.org/licenses/by/4.0/ This content is distributed under the terms of the Creative Commons Attribution 4.0 International license.
