# Supplementary figures and images for: Temporal Dynamics of Rare and Abundant Soil Bacterial Taxa from Different Fertilization Regimes Under Various Environmental Disturbances
Source: mSystems. 2022 Sep 19;7(5):e00559-22. doi: 10.1128/msystems.00559-22 (PMC9600180; doi:10.1128/msystems.00559-22)

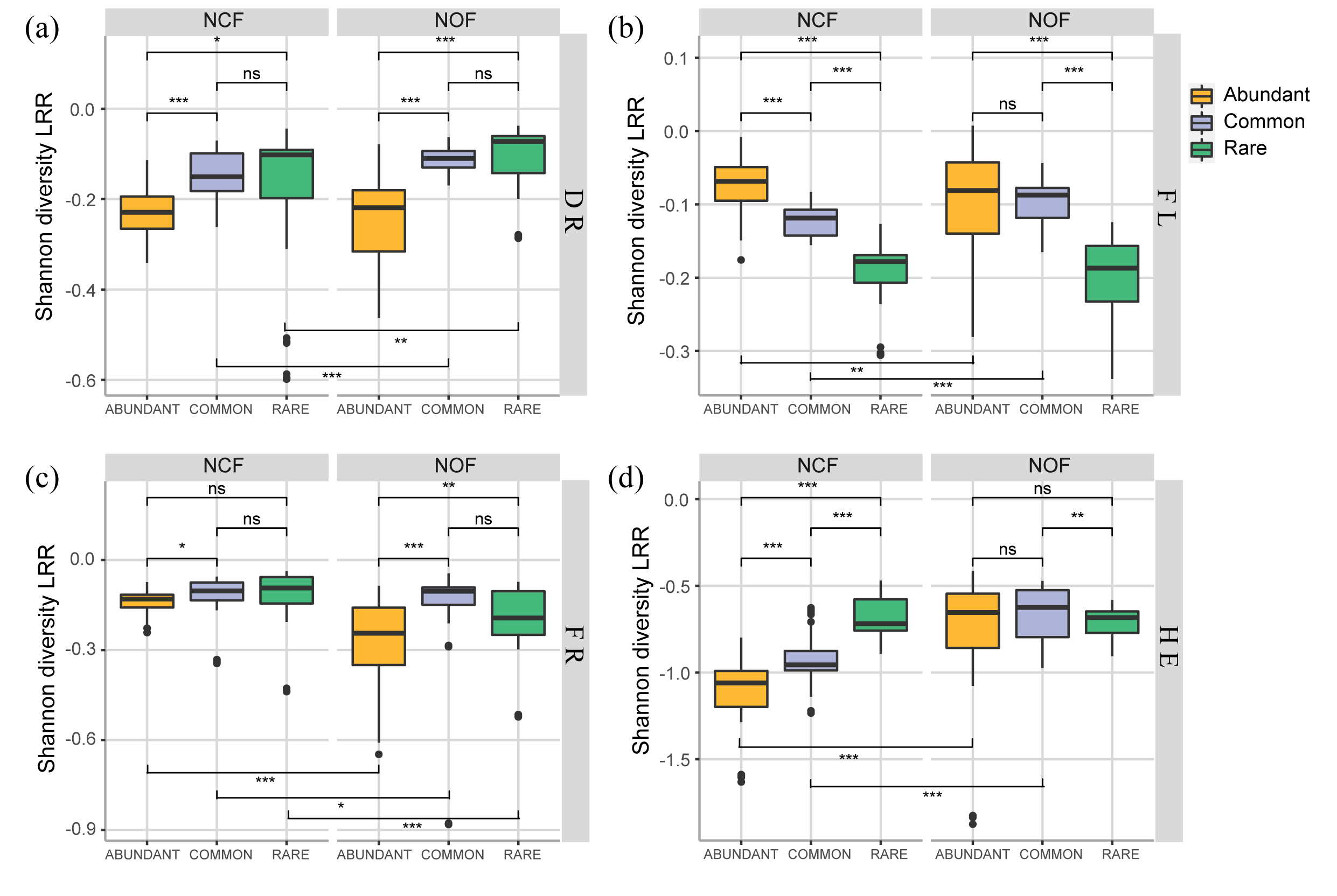

Supplement: FIG S1 [file msystems.00559-22-s0001.tif]

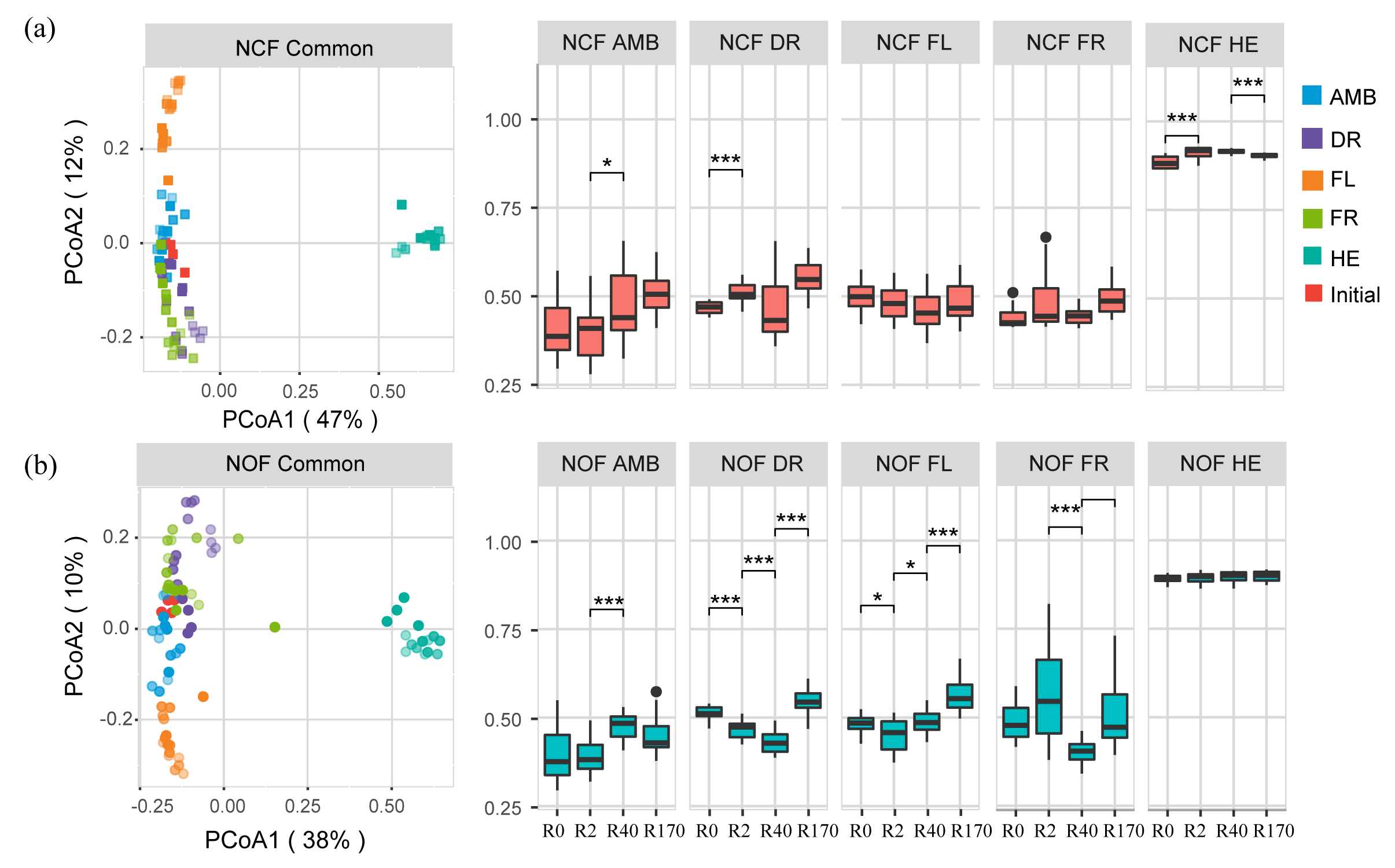

Supplement: FIG S2 [file msystems.00559-22-s0002.tif]

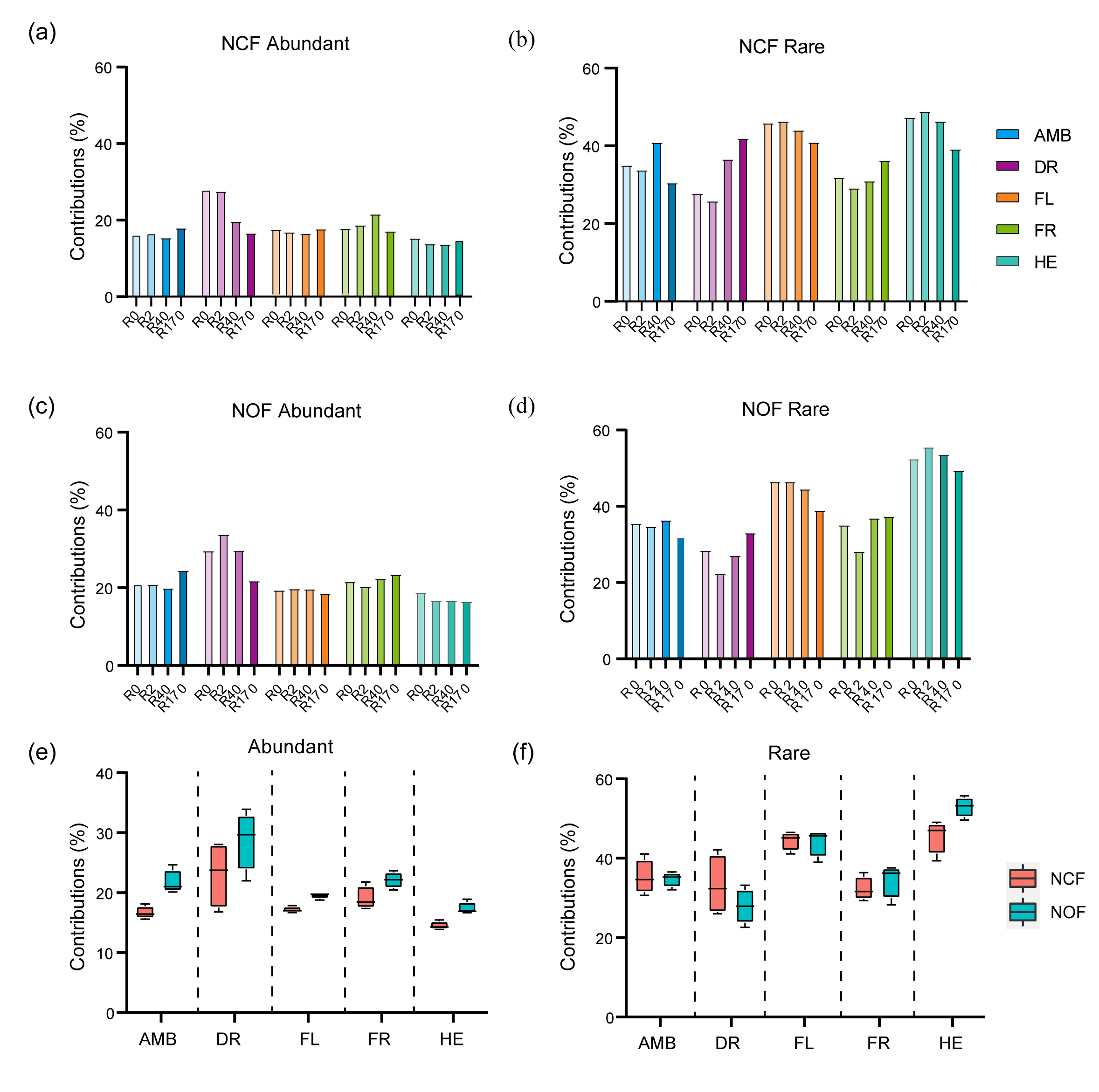

Supplement: FIG S3 [file msystems.00559-22-s0003.tif]

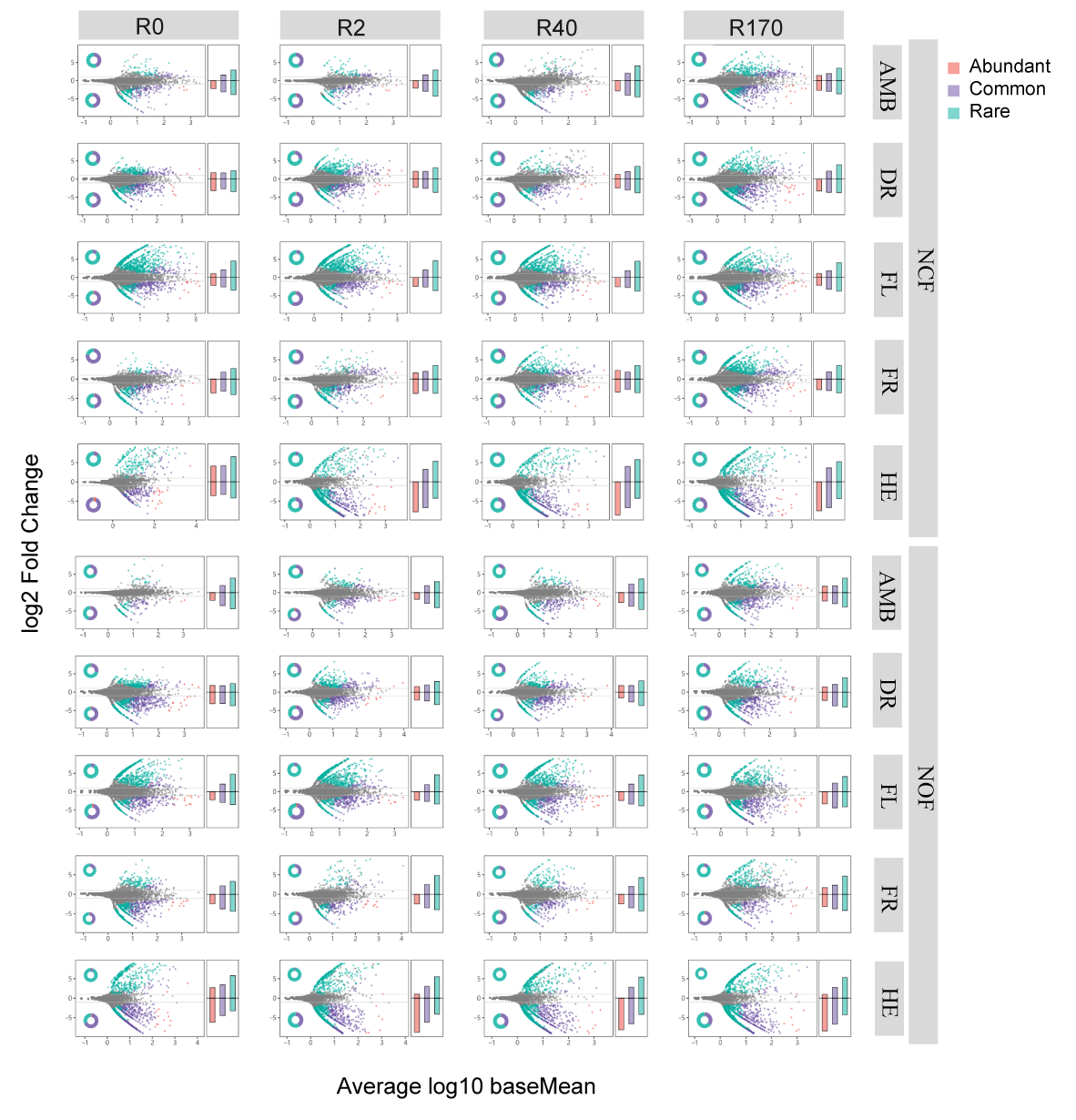

Supplement: FIG S4 [file msystems.00559-22-s0004.tif]

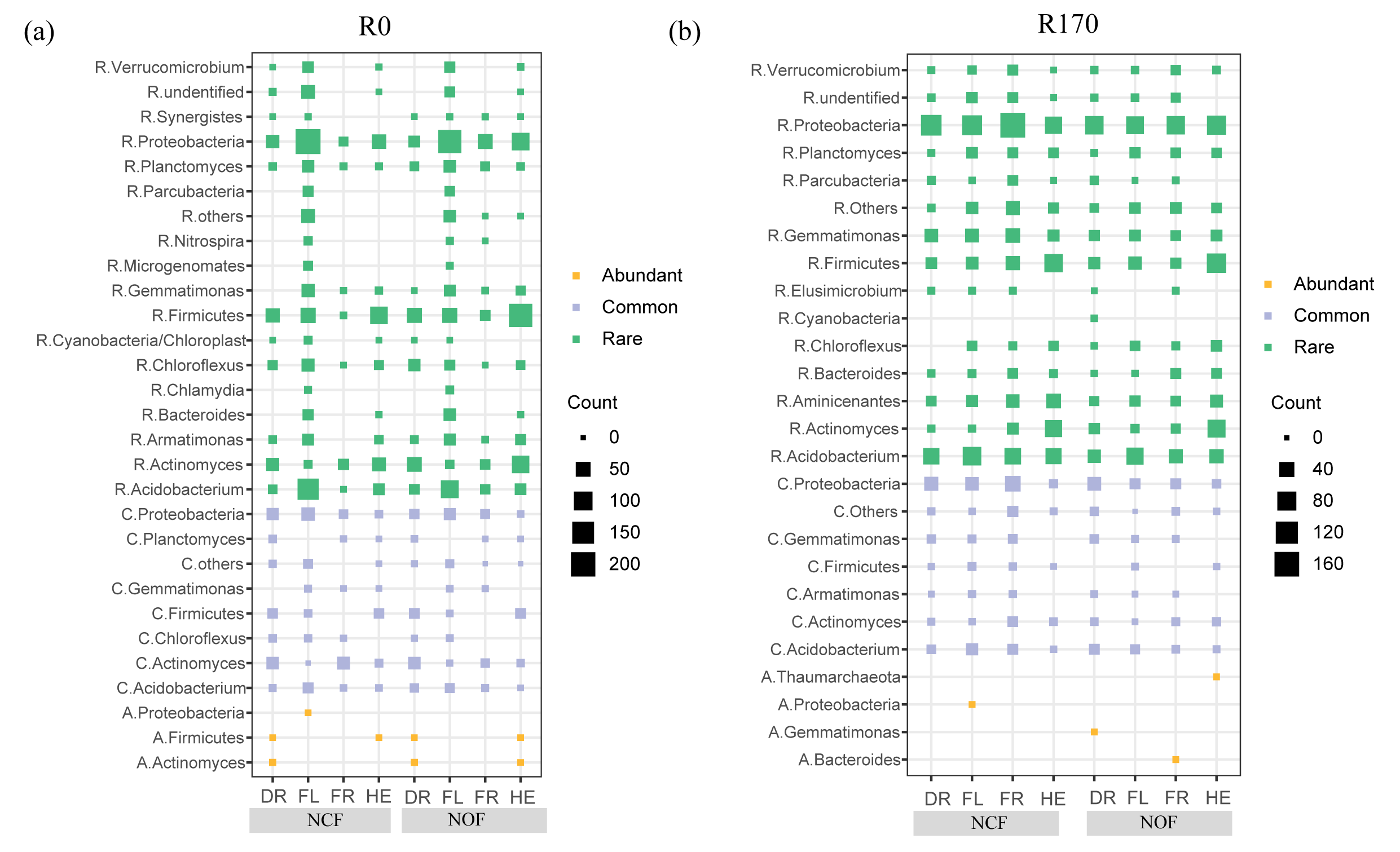

Supplement: FIG S5 [file msystems.00559-22-s0005.tif]

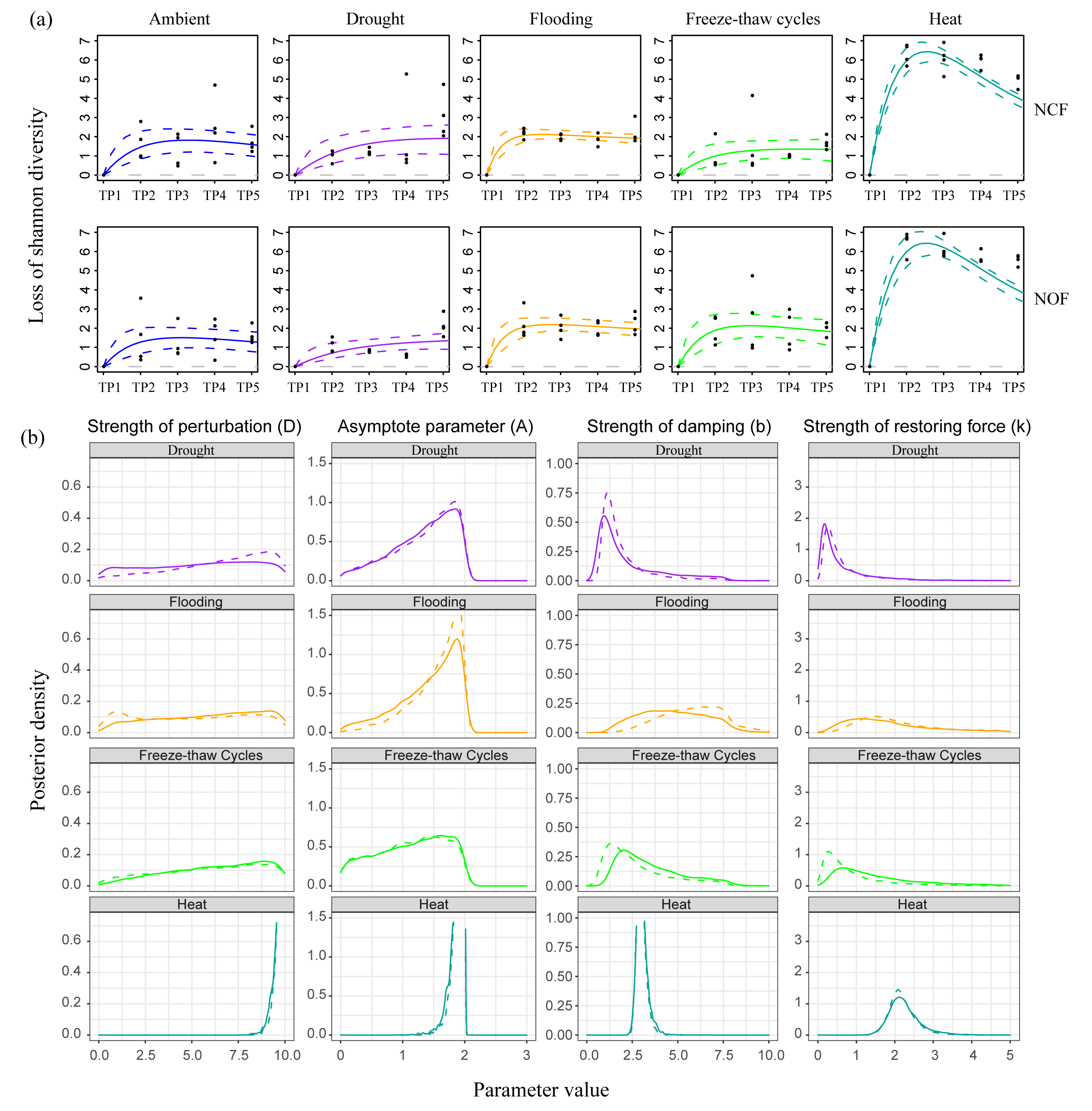

Supplement: FIG S6 [file msystems.00559-22-s0006.tif]
