# Supplementary material for: Temporal Dynamics of Rare and Abundant Soil Bacterial Taxa from Different Fertilization Regimes Under Various Environmental Disturbances
Source: mSystems. 2022 Sep 19;7(5):e00559-22. doi: 10.1128/msystems.00559-22 (PMC9600180; doi:10.1128/msystems.00559-22)
Supplement: TABLE S1 [file msystems.00559-22-s0007.docx]

| Soil type | Main test | Abundant | | Common | | Rare | |
| --- | --- | --- | --- | --- | --- | --- | --- |
|  |  | PERMANOVA | | PERMANOVA | | PERMANOVA | |
|  |  | R^2^ | *P* ( > F) | R^2^ | *P* ( > F) | R^2^ | *P* ( > F) |
| NCF | Disturbance | 0.61 | 0.001 | 0.67 | 0.001 | 0.45 | 0.001 |
|  | Time | 0.094 | 0.001 | 0.053 | 0.001 | 0.059 | 0.001 |
|  | Disturbance *Time | 0.13 | 0.001 | 0.12 | 0.001 | 0.15 | 0.001 |
| NOF | Disturbance | 0.54 | 0.001 | 0.54 | 0.001 | 0.43 | 0.001 |
|  | Time | 0.085 | 0.001 | 0.065 | 0.001 | 0.055 | 0.001 |
|  | Disturbance *Time | 0.16 | 0.001 | 0.16 | 0.001 | 0.15 | 0.001 |
