# Supplementary material for: Temporal Dynamics of Rare and Abundant Soil Bacterial Taxa from Different Fertilization Regimes Under Various Environmental Disturbances
Source: mSystems. 2022 Sep 19;7(5):e00559-22. doi: 10.1128/msystems.00559-22 (PMC9600180; doi:10.1128/msystems.00559-22)
Supplement: TABLE S2 [file msystems.00559-22-s0008.docx]

|  | | | | | | | | | | | | | | | | | | |  |
| --- | --- | --- | --- | --- | --- | --- | --- | --- | --- | --- | --- | --- | --- | --- | --- | --- | --- | --- | --- |
| Rare | |  | |  |  | D |  |  | | A |  |  | | Phi1 |  |  | | Phi2 |  |
| Microbiome | |  | Stress | |  | Media | 95%CI | |  | Media | 95%CI | |  | Media | 95%CI | |  | Media | 95%CI |
| NCF | |  | DR | |  | 7.13 | (1.56--9.76) | |  | 1.44 | (-0.08--1.96) | |  | -0.94 | (-1.67---0.10) | |  | 0.01 | (-0.73--1.50) |
|  | |  | FL | |  | 5.29 | (0.61--9.52) | |  | 1.65 | (0.66--1.97) | |  | -0.82 | (-1.46--1.21) | |  | 1.64 | (0.90--1.97) |
|  | |  | FR | |  | 6.20 | (1.02--9.66) | |  | 0.90 | (-0.99--1.89) | |  | -1.15 | (-1.90---0.03) | |  | 0.58 | (-0.61--1.82) |
|  | |  | HE | |  | 9.83 | (9.29--9.99) | |  | 1.94 | (1.75--2.00) | |  | 0.29 | (0.11--0.41) | |  | 0.47 | (0.34--0.74) |
| NOF | |  | DR | |  | 5.59 | (0.58--9.58) | |  | 1.38 | (-0.09--1.95) | |  | -1.26 | (-1.88---0.23) | |  | 0.10 | (-0.98--1.79) |
|  | |  | FL | |  | 6.21 | (1.08--9.70) | |  | 1.52 | (0.17--1.96) | |  | -0.85 | (-1.45--0.72) | |  | 1.39 | (0.31--1.94) |
|  | |  | FR | |  | 6.59 | (1.65--9.70) | |  | 0.96 | (-0.90--1.90) | |  | -0.68 | (-1.41--0.38) | |  | 0.86 | (-0.06--1.84) |
|  | |  | HE | |  | 9.81 | (9.18--9.98) | |  | 1.93 | (1.70--2.00) | |  | 0.29 | (0.08--0.42) | |  | 0.49 | (0.34--0.80) |
